# The delta high-density lipoprotein cholesterol ratio: a novel parameter for gram-negative sepsis

**DOI:** 10.1186/s40064-016-2685-4

**Published:** 2016-07-11

**Authors:** Guoying Zou, Junyu He, Biqiong Ren, Fei Xu, Guofeng Xu, Wenling Zhang

**Affiliations:** Department of Clinical Laboratory, Brain Hospital of Hunan Province, Furong Middle Road 427, Changsha, 410007 Hunan People’s Republic of China; Department of Medical Laboratory, Hunan University of Traditional Chinese Medicine, Changsha, People’s Republic of China; Department of Laboratory Medcine, Xiangya Medical School, Central South University, Changsha, People’s Republic of China

**Keywords:** Gram-negative sepsis, High-density lipoprotein, Bacterial infections

## Abstract

**Objective:**

To study changes in blood lipid metabolism in sepsis patients, especially high-density lipoprotein cholesterol (HDL-C) changes in the diagnosis of sepsis and the type of bacteria involved.

**Methods:**

Two-hundred-twenty cases of patients with febrile infections were divided into local infection, systemic inflammatory response syndrome or sepsis (sepsis) group. For controls, 81 cases of patients with a healthy check-up were used. Lipid levels and inflammatory state were supervised, and a comparative analysis of patients admitted to the hospital after 1, 5, 10 days was performed.

**Results:**

In patients with sepsis, total cholesterol, HDL-C, and apolipoprotein A 1 (apoA 1) were significantly decreased in this group. Particularly HDL-C was decreased 1 day after admission. Compared with the patients with gram-positive sepsis, HDL-C and apoA1 were significantly reduced in the patients with gram-negative sepsis at admission. The 24-h change ratio of HDL-C was different between the gram-negative and gram-positive sepsis patients with a 70.5 % specificity and 76.5 % sensitivity. The area under the curve was 0.744, and the critical value was −21.1 %.

**Conclusions:**

The sepsis patients had lower HDL-C than the other groups. The 24-h change ratio of HDL-C can be used as a sepsis diagnosis maker and to distinguish between the bacteria involved in sepsis.

## Background

A bacterial infection is an acute infection of growing pathogenic bacteria or conditional pathogenic bacteria that invade the blood circulation and produce toxins and other metabolites. Pathogenic bacteria can include gram-negative bacteria (gram^−^) and gram-positive bacteria (gram^+^). Bacterial infections can easily develop into sepsis in older children with poor immune systems if the infections are left untreated or have complications. Complications are regarded as the main reason for high morbidity and mortality. As the abuse of antibiotics and the difference of the immune system, the traditional diagnostic indicators for bacterial infection or sepsis are difficult to meet the clinical requirements in the matter of sensitivity and specificity, such as temperature, white blood count and erythrocyte sedimentation rate. Therefore, better biological indicators are needed for early diagnosis and prognosis.

Microbial cultures are the “gold standard” for diagnosing a bacterial infection. However, cultures can only yield results 48–72 h after receiving samples. Currently, even the results from a Gram stain require 12–24 h or longer. Although PCR can be a fast and reliable method for identifying bacteria for quality assurance (Hospodsky et al. 2010), its use is restricted in certain areas. Despite the availability of acute phase proteins, such as C-reactive protein (CRP), procalcitonin (PCT), and tumor necrosis factor, as sepsis markers, there is no ideal marker that can reliably differentiate between infected and non-infected patients. Lipoproteins and lipids, which have direct immunomodulatory properties, bind and neutralize toxic bacterial substances and could be new candidates as infection markers (Feingold et al. 1995). High-density lipoprotein (HDL), a lipoprotein that participates in the reverse transportation of cholesterol and selectively inhibits type I interferon gene responses, plays a role in regulating innate immune functions during bacterial infections (Suzuki et al. 2010). Apolipoprotein A 1 (apoA 1) is abundant in HDL and can combine a hydrophilic lipotropic alpha helix with lipid A endotoxin and can limit endotoxin activity, thus improving its bacterially toxic effects (Thaveeratitham et al. 2007).

The present study was designed to investigate changes in HDL level values or ratios in patients with sepsis. HDL can be checked by routine clinical testing (receiving results during 1 h) and is inexpensive and easy. We also aimed to determine the best threshold to distinguish gram^−^ sepsis infections from gram^+^ sepsis infections.

## Methods

### Study subjects

Between July 2012 and February 2013, study subjects were recruited from infectious inpatient clinics in the Brain Hospital of Hunan Province, China. The study was approved by the ethic committee of our hospital. During this period, a total of 220 subjects (123 males and 97 females) who had data regarding fever [we reviewed the each study patient suffered from high temperature (>38 °C)] or infection and were over the age of 18 were recruited. The febrile patients were devided into 3 groups. The patients of two groups were judged by the criteria for systemic inflammatory response syndrome (SIRS, n = 75) and sepsis (n = 76), as described by the 1991 American College of Chest Physicians/Society of Critial Care Medicine Consensus Conference (Bone et al. 1992). The study SIRS sujects met two or more of the following conditions without evidence of a bacterial infection: (1) temperature >38 °C; (2) heart rate >90 beats/min; (3) respiratory rate >20 breaths/min or PaCO_2_ < 32 mm Hg; (4) WBC count >12 × 10^9^/L, <4×10^9^/L or >10 % immature (bands) forms. The sepsis patients were diagnosed by the above-mentioned SIRS criteria and a bacterial infection evidence. The other group of patients with local infect (n = 69) only suffered from fever and confirmed infection without SIRS. The definitions for a microbiologically confirmed case of a bacterial infection included isolation of an organism from a culture from the blood (n = 49), urine at > 10^5^/mL (n = 18), needle aspiration of an abscess or empyema (n = 3), sputum sample (n = 68), or pharyngeal swab (n = 7). Microbiological evaluations confirmed bacterial infections (n = 145) in local infection and sepsis patients (Table 1).

**Table 1:** Grouping of patients with febrile infection

Group	Bacterial infection	Etiologic agents
Local infection (69)	Gram-positive (gram^+^) infection (27)	*Staphylococcus aureus* (6)
		*Streptococcus agalactiae* (12)
		*Streptococcus pyogenes* (3)
		*Streptococcus constellatus* (3)
		*Enterococcus faecium* (3)
	Gram-negative (gram^−^) infection (42)	*Escherichia coli* (9)
		*Klebsiella pneumoniae* (15)
		*Enterobacter cloacae* (3)
		*Enterobacter intermadium* (3)
		*Pseudomonas aeruginosa* (6)
		*Stenotrophomonas maltophilia* (3)
		*Acinetobacter baumannii* (3)
Sepsis (76)	Gram-positive (gram^+^) infection (33)	*Staphylococcus aureus* (27)
		*Staphylococcus epidermidis* (3)
		*Enterococcus faecium* (3)
	Gram-negative (gram^−^) infection (43)	*Moraxella lacunata* (2)
		*Acinetobacter baumannii* (11)
		*Klebsiella pneumoniae* (16)
		*Burkholderia* *cepacia* (2)
		*Escherichia coli* (5)
		*Pseudomonas aeruginosa* (3)
		*Stenotrophomonas maltophilia* (3)
		*Enterobacter aerogenes* (1)

Subgroups were sorted based on medical and microbiological examination, including isolation of an organism from a culture from the blood, urine, needle aspiration of an abscess or empyema, sputum sample or pharyngeal swab specimens. The number of presented cases was given in parentheses

Samples were selected from three cohorts: 69 cases of local infections (38 males, 31 females, age: 60.9 ± 21.1 years), 75 cases of systemic inflammatory response syndrome (41 males, 34 females, age: 56.3 ± 19.3 years), and 76 cases of sepsis (44 males, 32 females, age: 55.4 ± 17.6 years), with 33 cases of gram^+^ infection and 43 cases of gram^−^ infection. All subjects met a above-mentioned standard of sepsis diagnostics based on exclusion from one of the following: (1) recent infectious disease or chronic inflammatory disease; (2) serious hematologic disease; (3) bone marrow transplantation; (4) connective tissue disease or rheumatism; (5) application of inflammation-inhibiting drugs, such as a non-steroidal anti-inflammatory analgesic, steroid medicines, among others; (6) high cholesterol, high blood pressure, or diabetes; or (7) serious liver and/or kidney function insufficiency. A total of 81 serum samples from healthy volunteers were analyzed (47 males, 34 females, age: 52.6 ± 18.7 years). Ethics approval for the study protocol and data analysis was obtained from the committee of Brain Hospital of Hunan Province.

### Clinical and laboratory examination

Non-anticoagulant blood samples were taken at 0, 1, 5 and 10 days after admission to the hospital. Healthy volunteers provided samples only once. Total cholesterol (TCH), HDL-C, triglycerides (TG), apoA 1, apolipoprotein B (apoB), CRP and PCT were measured from blood samples. All measurements, except CRP and PCT, were performed using a Siemens ADVIA 2400 automatic biochemical analyzer with original Siemens reagents. CRP was measured using a NingBo MedicalSystem (China). PCT was tested with Roche cobas e411 (Switzerland). At the same time as the blood collection, all of the samples for bacterial culture from suspected infectious subjects were collected. Blood culture (BACTEC 9050, BD) and bacterial incubation (ChangSha ChangJin, China) and determination (Siemens microscan walkaway 40SI, Germany) were used to identify bacteria species.

### Statistical analysis

Descriptive data were reported as the mean-SD. Group differences were tested using analysis of variance (ANOVA). After ANOVA, pairwise group comparisons were performed using an LSD test with homogeneity of variance parameters and a Games-Howell inspection of the heterogeneity of variance parameters. A two-way factorial analysis of variance with repeated measures was used to quantify significant effects during a 10-day hospital admission. A post hoc analysis with a Bonferroni correction for multiple comparisons was also performed. The efficiency of each variable in differentiating between gram^+^ (n = 33) and gram^−^ (n = 43) sepsis was evaluated by a receiver operating characteristic (ROC) curve analysis. Based on this analysis, the cutoff values for each variable were determined to simultaneously maximize both the sensitivity and specificity percentages. In addition to sensitivity and specificity, a maximum area under the ROC curve (AUC) was calculated.

Graphpad Prism 5.0 (Graphpad Software, La Jolla, CA, USA) and SPSS 16.0 for windows (SPSS Inc., Chicago, IL, USA) were used for statistical analyses. All testing was two-tailed, and *p* values below 0.05 indicated statistical significance.

## Results

### Serum blood lipid levels and inflammatory state at admission

In our previous studies, we demonstrated that blood lipids change in patients with gram^−^ bacterial infections (Zou et al. 2005). However, we did not consider blood lipids to be a diagnostic for gram^−^ or gram^+^ sepsis. Here, we investigated whether the patients suffering from sepsis also had changes in blood lipids. We compared the patients with febrile infections with the healthy subjects at admission. We found the TCH was reduced in the patients with a local infection or SIRS (*p* < 0.001). CRP and PCT was increased (*p* < 0.001) and TCH, HDL-C and apoA1 (*p* < 0.001) were decreased in the sepsis patients. However, CRP was increased in the patients with a SIRS (*p* < 0.001), PCT was elevated in all febrile infectious patients (*p* < 0.001) (Table 2). The blood lipid levels changed in the febrile infectious patients in a state of inflammation. Specifically, TCH was inhibited. The above results showed that the blood lipid parameters changed at admission in all patient groups compared to controls, particularly the sepsis patients.

**Table 2:** Serum blood lipids level and inflammatory state at admission ($\bar{x}$ ± SD)

Group	Case (n)	TCH (mmol/L)	HDL-C (mmol/L)	TG (mmol/L)	apoA1 (g/L)	apoB (g/L)	CRP (mg/L)	PCT (ng/mL)
Local infection	69	3.74 ± 1.22**	0.91 ± 0.33	1.22 ± 0.74	0.95 ± 0.29	0.84 ± 0.22	54.3 ± 96.1**	0.21 ± 0.07**
SIRS	75	3.67 ± 1.22**	0.93 ± 0.31	1.17 ± 0.44	0.93 ± 0.25	0.80 ± 0.26	62.5 ± 67.5**	0.18 ± 0.10**
Sepsis	76	3.41 ± 1.06**	0.80 ± 0.31**	1.37 ± 0.84	0.83 ± 0.31**	0.83 ± 0.31	79.5 ± 94.0**	0.20 ± 0.12**
Control	81	4.62 ± 1.05	1.02 ± 0.22	1.39 ± 0.82	1.01 ± 0.16	0.94 ± 0.23	9.4 ± 17.4	0.04 ± 0.02

Data were expressed as mean values with standard deviation (SD). Patients and control group differences were tested using analysis of variance (ANOVA). After ANOVA, pairwise group comparisons were performed using an LSD test with homogeneity of variance parameters and a Games–Howell inspection of the heterogeneity of variance parameters

* p < 0.05; ** p < 0.01

### Serum blood lipid levels and inflammatory state at different times after admission

**Fig. 1:**
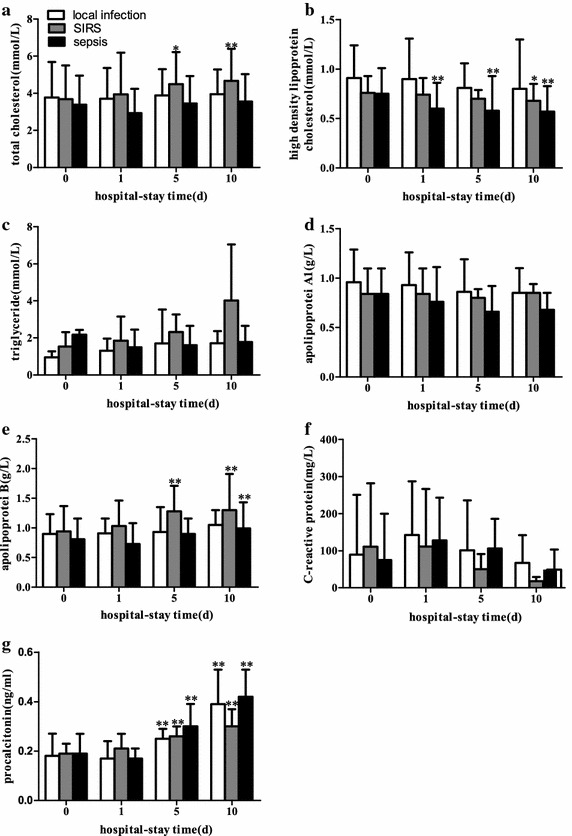
Serum lipid levels and inflammatory state at different times after admission. **a** Total cholesterol; **b** high-density lipoprotein cholesterol; **c** triglycerides; **d** apolipoprotein A1; **e** apolipoprotein B; **f** C-reactive protein; **g** procalcitonin. The *box chart* statistics (mean value and SD) of the data are also shown. Two-way factorial analysis of variance with repeated measures was used to quantify the main effects during the 10-day hospital admission. The post hoc analysis was performed using a Bonferroni correction for multiple comparisons

Because the blood lipid parameters changed at admission in the febrile infectious subjects, we next determined whether the blood lipid parameters had dynamic changes during hospitalization in our study subjects. By monitoring the levels of serum blood lipids at 0, 1, 5, 10 days after admission, we found that the febrile infectious subjects had differences in blood lipid levels, sch as TCH (*p* = 0.003), HDL-C (*p* < 0.001) and apoB (*p* < 0.001), and in inflammatory parameter PCT (*p* < 0.001). These levels also significantly changed during the course of the admission in different patients. The HDL-C levels had a tendency to only be reduced in the sepsis group and generally began to drop within 24 h of admission. The SIRS subjects had elevated TCH and apoB. PCT started to elevate within 5 days after admission in all febrile infectious patients (*p* < 0.001) (Fig. 1). These results illustrate that HDL-C was markedly suppressed in the sepsis patients during hospitalization.

### Serum blood lipid levels in the patients with gram^−^ sepsis at admission

**Fig. 2:**
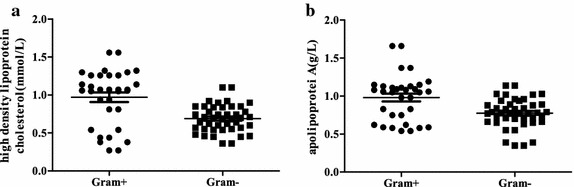
Influence of different Gram stain bacterial sepsis on the blood lipid levels at admission. **a** High-density lipoprotein cholesterol. **b** Apolipoprotein A1. The *box chart* statistics (mean value and SEM). The difference between the Gram^+^ and Gram^−^ sepsis groups were tested using an independent-samples T test

In this study, we observed that HDL-C was more greatly inhibited in gram^−^ sepsis. Although PCT can indicate patients’ inflammatory state, can’t distinguish different gram-stained type. From the analysis of the lipids levels of the sepsis patients with different gram-stained bacteria at admission, we found that compared with the patients with gram^+^ sepsis, HDL-C (*p* < 0.001) and apoA1 (*p* = 0.008) were significantly reduced in the patients with gram^−^ sepsis (Fig. 2; Table 3). The remaining blood lipid parameters were not different between the Gram classifications (Table 3).

**Table 3:** Serum blood lipids levels and inflammatory state in gram^−^ sepsis at admission ($\bar{x}$ ± SD)

Group	Case (n)	TCH (mmol/L)	HDL-C (mmol/L)	TG (mmol/L)	apoA1 (g/L)	ApoB (g/L)	CRP (mg/L)	PCT (ng/mL)
Gram^+^ sepsis	33	3.69 ± 0.99	0.97 ± 0.37	1.50 ± 1.59	0.98 ± 0.30	0.87 ± 0.35	69.9 ± 93.6	0.17 ± 0.10
Gram^−^ sepsis	43	3.14 ± 0.95	0.69 ± 0.18**	1.44 ± 0.93	0.78 ± 0.19**	0.78 ± 0.24	85.9 ± 95.2	0.24 ± 0.13

Data were expressed as mean values with standard deviation (SD). Gram^+^ (n = 33) and gram^−^ (n = 43) sepsis group differences were tested using independent-samples T test

* p < 0.05; ** p < 0.01

### Delta HDL-C ratio in detecting gram^−^ sepsis

The HDL-C and apoA1 levels had a tendency to be reduced in the sepsis patients. This decrease was more obvious in the gram^−^ sepsis patients. The largest decrease occurred on the sixth day after admission. Interestingly, in contrast to gram^+^ sepsis, gram^−^ sepsis induced a potent downregulation of HDL-C on the sixth day. We determined how much HDL-C had changed in the gram^−^ sepsis patients by calculating the ratio of the HDL-C decrease $\left[ \left( {\frac{{HDL{ - }C_{1d} - HDL{ - }C_{0 \, d} }}{{HDL - C_{0d} }}} \right) \times 100\;\%\right]$ and conducting a ROC curve analysis on the gram^−^ sepsis patients within 24 h of admission.

**Fig. 3:**
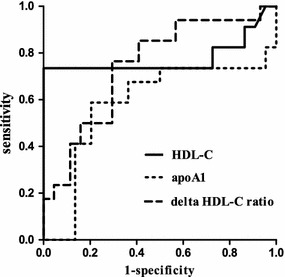
Receiver operating characteristic (ROC) curves comparing the serum HDL-C and apoA1 levels and the 24 h change ratio of HDL-C for distinguishing between gram^+^ sepsis (n = 33) and gram^−^ sepsis (n = 43). The statistics of the ROC curve analysis and the significance of pairwise comparisons between the areas under the ROC curve (AUC) are also shown

The ROC curve analysis confirmed that the delta ratio of HDL-C was different between the gram^−^ sepsis and gram^+^ sepsis patients and had a 70.5 % specificity and 76.5 % sensitivity. The area under the curve was 0.744 (SE = 0.057, *p* < 0.001). The highest observed value −21.1 % (Fig. 3; Table 4).

**Table 4:** Statistic of ROC curve analysis

	HDL-C	apoA1	Delta HDL-C ratio
Cutoff point	<0.86	<0.86	<−21.1 %
AUC (SE)	0.777 (0.065)	0.586 (0.072)	0.744 (0.057)
Sensitivity (%)	73.5	58.8	76.5
Specificity (%)	100.0	79.5	70.5
PPV (%)	100.0	67.9	65.8
NPV (%)	82.7	70.8	78.9
PLR	∞	2.87	2.59
NLR	0.27	0.52	0.42
*p* value	<0.001	0.197	<0.001

Data were expressed as the statistics of ROC curve analysis and the significance of pairwise comparisons between gram^+^ sepsis (n = 33) and gram^−^ sepsis (n = 43)

## Discussion

Because severe sepsis and septic shock can be fatal within hours, it is customary to start empirical broad-spectrum antimicrobial therapy in all patients hospitalized with the suspicion of systemic inflammatory response syndrome (Bochud et al. 2004), However, the overuse of broad-spectrum antimicrobials without bacterial identification has contributed to the emergence of drug-resistant strains of bacteria, especially in ICU patients. For example, first identifying the causative pathogen of an infection and then using an appropriate narrow-spectrum antibiotic against gram^+^ or gram^−^ bacteria should be a standard procedure to prevent antimicrobial overuse and resistance. However, microbial cultures are time consuming. Therefore, determining a new quick method to determine the type of infecting pathogen is essential.

We have proven in our previous studies that HDL-C and apoA 1 are significantly decreased in patients with a gram^−^ infection. Continuously low levels of HDL-C and apoA1 hint at an uncontrolled infection (Zou et al. 2006). In addition, patients with a gram^+^ infection have none of the above changes. Similarly, in this study, we found that blood lipids changed in the patients with all types of infections. TCH, HDL-C and apoA1 were significantly lower in the sepsis subjects. HDL-C appeared to gradually trend downward from admission after 1 day and was positively related to the severity of disease. As Kumaraswamy et al. (2012) demonstrated, HDL-C was greatly reduced in sepsis and SIRS, with the lowest HDL-C levels in sepsis shock. The degree of HDL-C reduction was associated with the severity of disease. We found in the SIRS group total cholesterol levels increased after day 5 and HDL-C did not change until day 10, this was not consistent with the finding of Kumaraswamy SB, which TCH and HDL-C were decrease. We observed different time period. The study test of Kumaraswamy SB was within 24 h. Moreover, there may be the treatment of the SIRS patients was effective during 10 day, thereby acute-phase proteins HDL-C did not fall, and the patients had good prognosis, so the every sick can survive.

In recent years, a large number of animal studies have found that HDL can combine with and neutralize the lipopolysaccharide (LPS) of gram-negative bacteria (Khovidhunkit et al. 2004). Lipoproteins bind with bacterial cell walls and cholesterol-loaded macrophages fight against LPS during bacterial infections. HDL selectively inhibits IFN I gene activity, thus playing a role inregulating innate immune function (Suzuki et al. 2010). Studies have shown that in vitro, pure LPS combines with 60 % HDL (Levels et al. 2001), and there are similar results with lipoteichoic acid (LTA) (Levels et al. 2003). This shows that HDL changes are related to gram^−^ bacterial infections.

In this study, we reported that decreased HDL-C levels correlate with the likelihood of gram^−^ bacterial infections, especially gram^−^ sepsis. HDL contains both proteins and cholesterol, but the cholesterol content is relatively stable and less influenced by the diet and nutritional status. The degree of variation of HDL-C was 0.7 %. For the first time, we were able to demonstrate a novel parameter for gram^−^ sepsis, the 24 h delta HDL-C ratio. Although the HDL-C ratio can’t be used until after 24 h after admission, which seemingly makes the time-difference to the gram stain method the same. The gram stain method can be implemented after cultivating bacteria, which may require 24 to 48 h, such as blood culture, in particular the slowly grown bacteria. The advantages of the delta HDL-C ratio over pathogen culture include that it is based on a routine measurement of HDL-C and is convenient, economical and assured quality. Trial specimen is more easy to get, collected conveniently. It takes less than an hour to receive HDL-C results, and the test can be performed quickly at any time without advanced technology.

In an in vitro experiment, HDL combined with bacterial LPS via an LPS binding protein, thereby reducing TLR4 expression and signal transduction (Van Linthout et al. 2011), down-regulating the NF-kappa B pathway and alleviating inflammation and mortality induced by LPS. Clinical studies by Van Leeuwen et al. (2003) have shown that lipoprotein levels are significantly decreased in ICU sepsis patients and are positively correlated with mortality. Gruber et al. (2009) demonstrated a similar phenomenon in lower respiratory tract infections. The CRP values decreased to 1.01 mg/dl and HDL-C to 0.18 mmol/L in the disease. The level of 0.82 mmol/L HDL-C was indicative of respiratory infections. The present study suggests that the blood lipid levels changed in the febrile infectious patients at admission. TCH, HDL-C and apoA1 decreased, particularly in sepsis patients (Table 2). Therefore, we surveyed the blood lipid parameter changes throughout hospitalization. The results demonstrated that HDL-C levels had a sustained decline starting at 24 h after admission in the sepsis patients, which was different from the SIRS and local infections (Fig. 1).

Sepsis developed quickly and led to a high mortality rate. Sepsis has often been treated with broad spectrum antibiotics, which has led to an emergence of resistant strains. Thus, first distinguishing between gram^+^ or gram^−^ sepsis is helpful in choosing the appropriate antibiotics for effective treatment. Thus, an accurate method is needed to effectively and quickly identify the types of pathogenic bacteria involved in sepsis. Recently, PCT and CRP can predict and identify patients for suspected sepsis in an emergency department (Tsalik et al. 2012). Futhermore, studies have reported signifi cantly higher levels in infections caused by gram-negative organisms than gram-positive or fungal infections (Brodska et al. 2013). However, another study showed that the PCT levels are higher in patients with *S. aureus* infection than in those with coagulase-negative staphylococci (Shomali et al. 2013). These indicated the PCT level is affected by a variety of infectious Bacterial species, so it is difficult to distinguish between gram staining sepsis, and we found PCT can’t distinguish between gram^−^ and gram^+^ sepsis (Table 3).

Berbee et al. (2005) found that in animals, HDL can combine with the cell walls of gram^−^ LPS and weaken the body’s biological reaction. They also found that HDL can protect rodents from death caused by sepsis. In this study, we tested the lipid levels in gram^+^ or gram^−^ sepsis patients and found that HDL-C was reduced within 1 day after admission in the gram^−^ sepsis patients (Table 3; Fig. 2). According to the ROC curve analysis, the delta ratio of HDL-C displayed a 70.5 % specificity and 76.5 % sensitivity for the detection of gram^−^ sepsis in the febrile patients with bacterial infections. The critical value was −21.1 % (Table 4). Studies have shown that gram stain of the knee aspirates was 45 % sensitivity and 100 % specificity in acute septic arthritis (Faraj et al. 2002), the sensitivity and specificity of Gram stain of liver abscesses aspirates were 52 and 94 % for gram-negative bacilli (GNB) and the sensitivity of the blood cultures for any GNB present were 39 % in liver abscesses patients (Chemaly et al. 2003). These show gram stain have lower sensitivity. In our retrospective study, most of objects hadn’t results of Gram stain before culture. So unfortunately we can’t compare the delta ratio of HDL-C with Gram stain before culture in gram^−^ sepsis. Interestingly, HDL-C had an 100 % specificity and 73.5 % sensitivity (Fig. 3), which was superior to the delta HDL-C ratio. Yildiz et al. (2009) also showed that in late onset neonatal sepsis, the HDL-C levels were significantly lower, with a 96.2 % specificity and 44.4 % sensitivity. However, there are other factors that influence the HDL-C levels, such as the nutritional status, liver status, lipid metabolism, and so on. Moreover, we studied retrospective, typical cases. Finally, we considered the change rate of HDL-C in gram^−^ bacterial sepsis patients. The 24-h time point sample was determined to be the best for observing an initial change. ApoA1 was not different between the gram^+^ and gram^−^ sepsis patients after 24 h. In summary, the delta decrease ratio was the most appropriate measurement to detect gram- sepsis compared with the individual variation in the serum HDL-C and apoA1 levels. Unfortunately, there still means that 23.5 % of the patients tested gram^+^ sepsis are actually gram^−^ sepsis and 29.5 % that are tested gram^−^ sepsis are actually gram^+^ sepsis. A research has shown that patients with septic shock associated with GNB infection can decrease the survival rate of patients 3.5-fold, survival fraction changes of 3.9-fold with inappropriate initial empiric therapy (Kumar et al. 2009). So it was not enough only with the HDL-C ratio distinguish between gram^−^ and gram^+^ sepsis. If a joint detection of the delta HDL-C ratio and HDL-C were used, the specificity would increase to 100 %, which will reduced the mortality of sepsis patients.

Patients with gram^−^ sepsis account for approximately 60 % (24/40)of patients with ICU sepsis. gram^−^ sepsis also has a long disease course. About 70 % of patients are still in a severe state five days after admission, which is noted by HDL-C being maintained at a low level. In the prognosis of our study subjects, two cases sepsis patients in vegetative state, sepsis prognosis of four cases were death after 10 days, while all patients survived in other groups. One departed gram^+^ sepsis patient, the 24 h HDL-C reduced 13.08 %, other three non-survivors were gram^−^ sepsis, the 24 h HDL-C decreased 25, 31.43, 50.59 %, respectively. HDL-C may have a protective effect against sepsis. In previous hospital studies, every HDL increase of 1 mg/mL reduced the occurrence rate of severe sepsis by 3 % (Grion et al. 2010). HDL levels above a critical value can indicate gram^−^ sepsis. These parameters can assist in determining the appropriate antibiotics to use for sepsis in a timely and effective manner, thus reducing antibiotic overuse and decreasing mortality.

No single method is dependable enough to confirm a fast etiologic diagnosis of sepsis. Instead, a final diagnosis should be determined by a doctor based on a sepsis diagnosis guide. The delta HDL-C ratio test could provide additional laboratory proof to rapidly and conveniently choose an appropriate antibiotic treatment in suspected sepsis.

## Conclusions

Sepsis patients had lower HDL-C. The 24-h HDL-C change ratio can be used as a novel sepsis diagnostic marker and as a convenient test to determine the type of bacteria involved in an infection. This information can be used to choose appropriate antibiotics and to quickly rescue patients, thereby reducing mortality.

## Abbreviations

HDL-C: high-density lipoprotein cholesterol
apoA 1: apolipoprotein A 1
TCH: total cholesterol
TG: triglycerides
apoB: apolipoprotein B
CRP: C-reactive protein
PCT: procalcitonin
LPS: lipopolysaccharide
Gram^−^: gram-negative bacteria
Gram^+^: gram-positive bacteria
SIRS: systemic inflammatory response syndrome
GNB: gram-negative bacilli
